# Integrating Drug Target Information in Deep Learning Models to Predict the Risk of Adverse Events in Patients with Comorbid Post-Traumatic Stress Disorder and Alcohol Use Disorder

**DOI:** 10.3390/biomedicines12122772

**Published:** 2024-12-05

**Authors:** Oshin Miranda, Xiguang Qi, M. Daniel Brannock, Ryan Whitworth, Thomas R. Kosten, Neal David Ryan, Gretchen L. Haas, Levent Kirisci, Lirong Wang

**Affiliations:** 1 Department of Pharmaceutical Sciences, School of Pharmacy, University of Pittsburgh, Pittsburgh, PA 15213, USA; osm7@pitt.edu (O.M.); xiq24@pitt.edu (X.Q.); levent@pitt.edu (L.K.); 2 RTI International, Durham, NC 27709, USA; mbrannock@rti.org (M.D.B.); rwhitworth@rti.org (R.W.); 3 Menninger Department of Psychiatry, Baylor College of Medicine, Houston, TX 77030, USA; kosten@bcm.edu; 4 Department of Psychiatry, School of Medicine, University of Pittsburgh, Pittsburgh, PA 15213, USA; nryan@pitt.edu (N.D.R.); haasgl@upmc.edu (G.L.H.); 5 Department of Psychology, Dietrich School of Arts and Sciences, University of Pittsburgh, Pittsburgh, PA 15260, USA; 6 VISN 4 Mental Illness Research, Education and Clinical Center (MIRECC), VAA Pittsburgh Healthcare System, Pittsburgh, PA 15240, USA

**Keywords:** post-traumatic stress disorder, drug targets, alcohol use disorder, artificial intelligence, beneficial medications

## Abstract

**Background/Objectives:** Comorbid post-traumatic stress disorder (PTSD) and alcohol use disorder (AUD) patients are at a significantly higher risk of adverse outcomes, including opioid use disorder, depression, suicidal behaviors, and death, yet limited treatment options exist for this population. This study aimed to build on previous research by incorporating drug target information into a novel deep learning model, T-DeepBiomarker, to predict adverse outcomes and identify potential therapeutic medications. **Methods:** We utilized electronic medical record (EMR) data from the University of Pittsburgh Medical Center (UPMC), analyzing 5565 PTSD + AUD patients. T-DeepBiomarker was developed by integrating multimodal data, including lab results, drug target information, comorbidities, neighborhood-level social determinants of health (SDoH), and individual-level SDoH (e.g., psychotherapy and veteran status). The model was trained to predict adverse events, including opioid use disorder, suicidal behaviors, depression, and death, within three months following any clinical encounter. Candidate medications targeting significant proteins were identified through literature reviews. **Results:** T-DeepBiomarker achieved high predictive performance with an AUROC of 0.94 for adverse outcomes in PTSD + AUD patients. Several medications, including OnabotulinumtoxinA, Dronabinol, Acamprosate, Celecoxib, Exenatide, Melatonin, and Semaglutide, were identified as potentially reducing the risk of adverse events by targeting significant proteins. **Conclusions:** T-DeepBiomarker demonstrates high accuracy in predicting adverse outcomes in PTSD + AUD patients and highlights candidate drugs with potential therapeutic effects. These findings advance pharmacotherapy for this high-risk population and identify medications that warrant further investigation.

## 1. Introduction

Post-traumatic stress disorder (PTSD) often co-occurs with substance use disorders (SUDs), including alcohol use disorder (AUD) [1]. Both conditions have specific diagnostic criteria. According to the DSM-IV and DSM-5, a PTSD diagnosis requires exposure to a significant stressor; an immediate emotional reaction involving fear, horror, or helplessness; and persistent symptoms, including re-experiencing the trauma, avoidance of related stimuli, heightened arousal, and significant impairment in social or occupational functioning lasting for at least one month. Similarly, AUD, as defined in the DSM-IV and DSM-5, involves maladaptive patterns of behavior linked to chronic substance use [2,3]. Over time, the clinical definition of PTSD has evolved across successive editions of the International Classification of Diseases (ICD). In the ICD-9, PTSD is characterized by symptoms such as re-experiencing the trauma, avoiding related stimuli, and heightened arousal following a psychologically traumatic event. The ICD-10 expanded on this by requiring the trauma to be “exceptionally threatening or catastrophic” in nature, with symptoms such as flashbacks, nightmares, and hyperarousal typically emerging within six months. The ICD-11 further refines this definition into three core symptom clusters: re-experiencing the trauma in the present (e.g., flashbacks or intrusive memories), avoidance of trauma-related triggers, and a persistent sense of heightened threat, including hypervigilance. These symptoms must significantly impair functioning and persist for weeks after the trauma. This progression highlights increasing specificity in the symptom descriptions, functional impacts, and timeframes for diagnosis. While a significant percentage of the U.S. population (50–89%) is exposed to trauma, only a small portion (6–8%) develops PTSD [4]. The susceptibility to PTSD is influenced by factors such as socio-demographics, co-occurring mental health conditions, and genetic predisposition [5]. Notably, approximately one-third of individuals with PTSD also meet the criteria for alcohol dependence, which may arise from multiple factors. Clinically, alcohol is often used to cope with PTSD symptoms, and genetic vulnerabilities may overlap between the two conditions [6]. Furthermore, individuals with a family history of alcohol dependence appear to have a higher likelihood of trauma exposure and PTSD development [7,8]. Identifying shared genetic markers for PTSD and AUD could pave the way for more targeted and personalized therapeutic approaches.

Pharmacological treatments for co-occurring PTSD and AUD have shown mixed outcomes. Medications such as sertraline, naltrexone, disulfiram, topiramate, and paroxetine have been studied, with varying results. For instance, sertraline has shown potential to reduce PTSD symptoms, although it did not significantly impact alcohol consumption [9]. Naltrexone and disulfiram, individually or in combination, have reduced drinking days and increased abstinence, with disulfiram also providing a modest PTSD symptom improvement [10]. Topiramate has demonstrated efficacy in reducing PTSD symptoms among combat veterans but did not affect alcohol use [11]. A comparison of paroxetine and desipramine, combined with naltrexone versus a placebo, showed no significant differences in PTSD symptom reduction between the two drugs; however, desipramine was associated with fewer heavy drinking days, although the lack of a placebo group complicates interpretation [12]. These results emphasize the need for further research to identify more effective medication options for these individuals.

Electronic medical records (EMRs) play a vital role in clinical documentation but often lack detailed data on drug targets. Recent research indicates that integrating multimodal data from EMRs, such as diagnostic information, medication usage, and lab results, can enhance predictions of high-risk patient outcomes [13,14,15,16,17]. Leveraging EMRs to identify evidence-based interventions enables more personalized treatment strategies, especially when standard therapies are ineffective. Advanced analytical tools, including deep learning and data mining algorithms, can uncover hidden patterns and relationships within large EMR datasets. These techniques can facilitate the discovery of beneficial medications and drug targets. In this study, the T-DeepBiomarker model was utilized to predict adverse event outcomes in PTSD and AUD high-risk cohorts. The insights derived from this model highlight potential medications and drug targets, laying the foundation for improved treatment strategies and the prevention of adverse outcomes.

## 2. Materials and Methods

### 2.1. Source of Data

This study utilized data from the Neptune system at the University of Pittsburgh Medical Center (UPMC), spanning January 2004 to October 2020 (https://www.dbmi.pitt.edu/services/; accessed on 25 March 2024). The Neptune system offers a collection of multimodal real-world data (e.g., demographic information, medical diagnosis information, prescribed medications, and laboratory test results). Patients were identified based on specific ICD-9 and ICD-10 codes and not DSM-IV or DSM-5 (detailed in Appendix A). The inclusion of multiple ICD versions (ICD-9 and ICD-10) reflects the transition in coding standards during the data collection period (2004–2020). The ICD-9 codes were used prior to October 2015, after which the ICD-10 codes were adopted. Our goal was to ensure consistency and reliability in case identification [15,16].

### 2.2. Data Preparation

**Figure 1:**
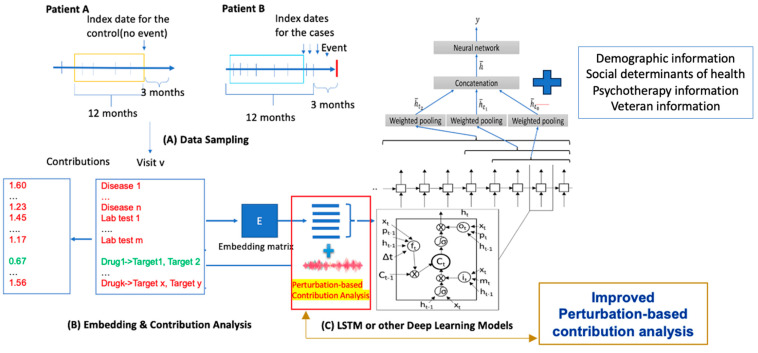
Our study workflow using T-DeepBiomarker. The workflow is organized into three main steps. (**A**) Data sampling from electronic medical records (EMRs): Patients meeting the inclusion criteria within the specified timeframe are identified. For example, Patient A, who experiences no adverse events, is classified as a control, while Patient B, who experiences at least one adverse event, is categorized as a case. Multimodal data—including diagnoses, medication use, social determinants of health (e.g., veteran status and psychotherapy), and lab test results or drug target information—are extracted from their structured EMRs to serve as input for the model. (**B**) Data embedding: The extracted multimodal data are transformed into continuous vectors to create an embedding matrix that captures the relationships between features. (**C**) Prediction using neural networks: Advanced neural network architectures, such as Time-Aware Long Short-Term Memory (TLSTM), a type of LSTM and the Reverse Time Attention Model (RETAIN), are employed as the core prediction components. The model generates a comprehensive set of biomarkers and employs a perturbation-based contribution analysis to determine the relative contribution (RC) of each feature. Biomarkers with RC values greater than 1 are considered indicators of high risk, whereas those with RC values less than 1 are classified as low risk indicators.

The primary goal was to predict the occurrence of adverse events within a three-month window from any clinical encounter (referred to as the index date) for patients diagnosed with both PTSD and AUD (Figure 1).

#### 2.2.1. Definition of PTSD and AUD

PTSD: Diagnosed cases were those meeting the ICD-9 or ICD-10 criteria for PTSD.

AUD: Diagnosed cases adhered to the ICD-9 or ICD-10 definitions for AUD.

For co-occurring PTSD and AUD patients, diagnoses for both conditions needed to occur within a 12-month timeframe.

To define “recently diagnosed PTSD”, patients were required to meet the ICD-9/10 diagnostic criteria for PTSD and have an adverse event recorded within three months of the initial PTSD diagnosis. Those without such an event in this timeframe were excluded from the PTSD cohort.

#### 2.2.2. Definition of Adverse Events

Adverse events were identified based on the ICD-9 and ICD-10 codes outlined in Appendix A. Patients experiencing adverse events within three months of the index date were designated as “cases”, while those without such records during the same period were classified as “controls”. This stringent classification ensured clear distinction between cases and controls, although the study acknowledges as a limitation that some individuals may experience adverse events without progressing to PTSD.

#### 2.2.3. Augmentation Techniques

To enhance the sample size, additional patients who experienced new adverse events following their initial PTSD and AUD diagnoses were included. Patient histories spanning three months before the adverse event were also reviewed to capture relevant data (eligible encounters). For the control group, a similar number of encounters without PTSD or AUD diagnoses were randomly selected (“yoked” controls). To further expand the cohort, 3546 additional patients were incorporated. These individuals had prior adverse events at or before the time of both diagnoses but developed new types of adverse events after the index date.

The dataset included multimodal variables, such as diagnostic codes, standardized medication data (mapped to unique DrugBank IDs), lab results (classified as “ABNORMAL”, “HIGH”, or “LOW”), SDoH, psychotherapy details, veteran-specific information, and drug target data. Diagnoses were grouped into categories, and medications were standardized into DrugBank IDs for analysis. This information was structured into sequences to facilitate a comprehensive evaluation based on the disease categories, DrugBank IDs, and lab test outcomes.

### 2.3. Dataset Splitting

The dataset was partitioned using an 8:1:1 ratio (into training, validation, and test subsets, respectively), ensuring balanced representation for model training and evaluation.

### 2.4. Drug Target Dataset Collection

Building on prior analyses [15,16], this study utilized a refined approach to assess the impact of medications by focusing on drug targets rather than individual drugs. This strategy involved pooling medications with shared targets to increase the sample sizes, thereby enhancing the statistical power and providing insights into the molecular mechanisms. Approach: Drug target information was obtained from the DrugBank database. Each medication’s targets were identified, allowing for the substitution of drugs with their targets in the analysis. For example, sertraline—a medication approved for PTSD—acts on specific protein targets such as the sodium-dependent serotonin transporter, sodium-dependent dopamine transporter, and sigma receptor. In the analysis, these targets replaced sertraline to explore their individual contributions to adverse outcomes. Relative Contribution Analysis: Using a perturbation-based approach, this study examined the effects of modulating individual drug targets on the risk of adverse outcomes. This method revealed medications capable of influencing these targets, providing a foundation for the further exploration of their therapeutic potential for PTSD and AUD. Insights from this analysis were supported by a comprehensive review of the scientific literature to identify promising treatment options. This approach represents a shift toward leveraging molecular-level insights to improve the understanding of PTSD and AUD and optimize the treatment strategies.

### 2.5. T-DeepBiomarker

We employed the Pytorch_EHR platform, developed by the Zhi Group, to analyze and predict clinical outcomes using advanced recurrent neural networks, including TLSTM and RETAIN [18,19]. The Long Short-Term Memory (LSTM) network, a type of recurrent neural network, is particularly effective in identifying long-range dependencies in sequential data by leveraging its input, forget, and output gates to manage the information flow. Typically, it uses a sigmoid activation function for these processes. RETAIN, on the other hand, optimizes the analysis of electronic medical records (EMRs) by incorporating an attention mechanism, which assigns greater importance to critical events in a patient’s medical history while accounting for temporal relationships. Building on our previous DeepBiomarker frameworks, we adapted this approach to our current dataset. The input data included a range of multimodal factors, such as lab test results, medication records, social determinants of health, drug targets, and diagnostic information. To evaluate the relative contributions (RCs) of the identified factors, we applied a perturbation-based method, where variables were randomly altered to observe their impact on the model’s predictions. The framework was implemented using consistent parameter settings to maintain comparability with earlier versions [20]. These settings included an embedding dimension of 128, a hidden layer size of 128, a dropout rate of 0.2, eight network layers, and a patience value of three epochs for early stopping. Model performance was rigorously assessed through ten iterations of cross-validation, and the standard deviation of the results was calculated to ensure reliability.

### 2.6. Statistical Analysis

#### Assessment of Biomarker Importance for Prediction of Adverse Events

To evaluate the impact of clinical factors on the prediction of adverse events, we calculated the relative contribution (RC) of each feature. This metric was derived by dividing the median feature contribution during events (FC with event) by the median feature contribution during non-events (FC without event). Perturbation-based methods were employed to estimate these values. The RC values, along with their statistical significance, are summarized below, where FC denotes the contribution of a given feature:

$$RC\;value=\frac{{median(FC}_{with\;event})}{{median(FC}_{without\;event})}$$

$$RC\;significance=Wilcoxon\;rank\;sum\;test\;p\;value\;({FCs}_{with\;event}\;and\;{FCs}_{without\;event})$$

The FC value quantifies the overall impact of a feature for a given patient, accounting for multiple occurrences of the same feature. Since FC distributions are often non-normal, medians were used instead of means to compute the RC. Statistical significance was determined using *p*-values from Wilcoxon rank-sum tests, which compared the median FCs between events and non-events. We applied the false discovery rate (FDR), with a threshold of 0.05 for significance, ensuring a controlled proportion of false positives among the significant results. To enhance the analysis, the FC values were normalized and the RC values were scaled across all features. Normalization was achieved by calculating the ratio of each feature’s contribution to the total contributions, facilitating fair comparisons across patients with varying numbers of clinical encounters. Scaling was performed using the PTSD diagnosis as a reference, assigning it an RC of 1, thereby standardizing the RC values for other features. This method minimized the biases stemming from differences in visit frequencies between cases and controls, enabling the effective identification of high- and low-risk indicators. As the interpretability in model analysis evolves, more advanced techniques may further refine the insights. The validity of this approach was confirmed through the integration of prior knowledge, leading to the identification of novel patterns and potential drug candidates for future exploration.

We evaluated the model performance using the area under the receiver operating characteristic curve (AUROC).

## 3. Results

### 3.1. Strong Performance Demonstrated by T-DeepBiomarker

Table 1 summarizes the performance outcomes of three predictive models—RETAIN + SDoH, LR + SDoH, and TLSTM + SDoH. These models combine machine learning and deep learning approaches to predict adverse events of interest, differing primarily in their complexity and their ability to handle sequential and time-sensitive data. Logistic regression (LR) is a conventional linear model that assumes a straightforward relationship between the input variables and the outcome. While it is simple and interpretable, its capacity to capture complex patterns is limited. In contrast, LSTM excels in analyzing sequential data by using memory cells to retain information over time, making it suitable for tasks involving temporal dependencies. TLSTM builds upon LSTM by integrating time intervals between events, enabling it to efficiently identify time-sensitive patterns. RETAIN, or the Reverse Time Attention Model, takes a different approach by emphasizing the significance of specific past events through an attention mechanism. By analyzing key moments in reverse chronological order, RETAIN is particularly effective for healthcare data, where certain historical events disproportionately impact outcomes. These advanced models outperform LR, particularly in their ability to manage complex and time-dependent clinical information. Our results demonstrated that TLSTM and RETAIN consistently achieved high performance, with AUC scores ≥ 0.90, surpassing the LR + SDoH model across all metrics, including the AUC, precision, and F1-score. The integration of social determinants of health (SDoH) further enhanced the predictive power of the deep learning models, highlighting their ability to capture intricate patterns in clinical data and deliver more accurate predictions.

### 3.2. Identification of Beneficial Drugs and Their Associated Drug Targets for Prediction of Adverse Events

We employed a perturbation-based estimation approach to assess the relative importance of different input features in forecasting adverse events. The primary findings, summarized in Table 2, highlight the most influential medications with an RC < 1, identifying them as low-risk indicators or potentially beneficial treatments. Other inputs, such as lab test results, diagnoses, and social determinants of health, were excluded from this presentation as their patterns aligned with the findings from previous studies. Our focus instead centered on the newly identified medications and drug targets.

Table 2 highlights several drug targets that could influence the prediction of adverse events in individuals with PTSD and AUD. Acetylserotonin O-methyltransferase (ASMT), involved in melatonin synthesis, may help to regulate disrupted sleep–wake cycles in PTSD, potentially alleviating symptoms [21]. Peripherin (PERE), a neuron-specific intermediate filament, plays a crucial role in neuronal structure and function, with alterations possibly affecting PTSD- and AUD-related neural pathways [22]. Nuclear receptor ROR-beta (RORB) influences circadian rhythms, which are critical for mood and anxiety regulation in this population [23]. Cannabinoid receptors 1 and 2 (CNR1 and CNR2) modulate neurotransmitter release and have been linked to stress responses and addiction [24]. Voltage-dependent calcium channel subunit alpha-2/delta-3 (CA2D3) regulates calcium influx, affecting neurotransmission and potentially impacting PTSD and AUD symptoms [25]. Vitamin D-binding protein (VTDB) influences vitamin D availability, with higher levels associated with the risk of depression, while increased serum vitamin D may reduce stress [26]. Sphingomyelin phosphodiesterase (ASM) affects sphingolipid metabolism, influencing neuroinflammation in PTSD and AUD [27]. Synaptosomal-associated protein 25 (SNP25) is essential for neurotransmitter release and synaptic plasticity, impacting neural pathways in both conditions [28]. RhoB (RHOB) regulates cell survival and apoptosis, with dysregulation potentially affecting neuroplasticity [29]. Integrin alpha-V (ITAV) influences cell adhesion and signaling, impacting brain plasticity and stress responses [30]. Cadherin 11 (H3BUU9) affects cell adhesion in the central nervous system, influencing neural connectivity in PTSD and AUD [31]. Metabotropic glutamate receptor 5 (GRM5) modulates excitatory neurotransmission and synaptic plasticity [32]. Glucagon-like peptide 2 receptor (GLP2R) influences gastrointestinal function and stress responses [33]. Tubulin beta-5 chain (TBB5) and microtubule-associated protein 4 (MAP4) maintain the cytoskeletal integrity, influencing neuroplasticity [34]. Orexin receptor type 1 (OX1R) regulates arousal and wakefulness, which are often disrupted in PTSD and AUD [35]. Myosin-2 (MYP2) is involved in cellular movement and structure, affecting neural connectivity [36]. The cystine/glutamate transporter (XCT) regulates glutamate levels and oxidative stress, both important in PTSD and AUD [37]. Finally, the glucagon-like peptide 1 receptor (GLP1R) influences glucose metabolism and neuroprotection, impacting neural health [38]. These findings suggest that understanding these drug targets could lead to more targeted interventions, reducing adverse events in this patient population.

## 4. Discussion

To improve the performance of T-DeepBiomarker in predicting adverse event risks for patients with PTSD and AUD, we incorporated a wide range of features. In order to better understand the specific medications and their associated drug targets, as well as the mechanisms linking PTSD and AUD with related adverse events, we have identified a selection of key medications that may play a crucial role in enhancing adverse event prediction.

### 4.1. Impact of Medications on Prediction of Adverse Events in PTSD + AUD Patients

#### Potential Beneficial Medication Options

OnabotulinumtoxinA (Botulinum Toxin A): Botulinum toxin A (BTX-A), widely used in treating neuromuscular disorders, has demonstrated potential in reducing neuropathic pain by inhibiting neurotransmitter release [39]. Randomized controlled trials have shown decreased opioid use and improved sleep durations in patients, suggesting that BTX-A may be a valuable treatment option for these conditions [40].

Dronabinol: Dronabinol significantly alleviated opioid withdrawal symptoms during inpatient treatment compared to a placebo (*p* = 0.006), although it did not influence the initiation or retention rates of XR-naltrexone treatment.

Acamprosate: Acamprosate is a medication approved for the prevention of relapse in alcohol dependence, demonstrating significant efficacy, safety, and tolerability over time. It has been shown to improve abstinence rates in detoxified alcohol-dependent patients in most European studies, primarily through its action on N-methyl-D-aspartate (NMDA) receptors. Despite its moderate efficacy, Acamprosate remains an important treatment option for addiction. However, U.S. studies, such as the Alcohol Collaborative Study, have shown less success, and adherence to the medication remains low [41].

Celecoxib: Substance cravings are a frequent challenge for patients undergoing opioid detoxification. A study comparing Celecoxib to ibuprofen in reducing pain and opioid cravings in 32 detoxifying patients found that both drugs reduced pain, but only Celecoxib significantly decreased opioid cravings. Celecoxib was more effective than ibuprofen in curbing cravings, although both medications equally alleviated pain [42].

Melatonin: Melatonin is a promising therapeutic agent for patients with PTSD and AUD, addressing both the neurological and physiological effects of alcohol abuse. It regulates circadian rhythms and provides antioxidant, anti-inflammatory, and immunomodulatory effects, which may alleviate alcohol-induced neurological disorders like polyneuropathy and psychosis. Sleep disturbances, common in PTSD + AUD patients, increase the relapse and suicide risk, but Melatonin can help to manage sleep issues, reduce inflammation, and protect against alcohol-related liver and mitochondrial damage. Clinical trials have suggested that Melatonin may improve sleep quality in AUD patients [43]. An analysis revealed no notable differences between the groups, indicating the need for additional research on higher doses to thoroughly evaluate the therapeutic potential of Melatonin in this setting. While Melatonin helps to regulate sleep by signaling the onset of sleep, orexin-2 receptor agonists work by enhancing wakefulness and reducing symptoms associated with sleep disorders and potentially improving mental health. OX2R agonists such as Danavorexton [44], Firazorexton [45], SB-668875 [46], Suntinorexton [47], TAK-861, and TAK-994 [48] may provide a more targeted approach for the chronic sleep disorders seen in our patient cohort; however, they are poorly understood but have indications in narcolepsy, sleep architecture in old age, anesthesia emergence, opioid-induced sedation, respiratory depression, and even anti-tumoral properties and need further investigation regarding their effects on the PTSD population.

Verapamil: Verapamil, a calcium channel blocker, has shown promise in reducing alcohol consumption in animal studies by modulating the neurotransmitters involved in alcohol craving. In alcohol-dependent subjects, intravenous Verapamil has been shown to impact cardiovascular symptoms during withdrawal. While its impact on humans remains unclear, Verapamil’s potential in managing the cardiovascular symptoms linked to alcohol dependence warrants further investigation [49,50,51].

Ergocalciferol and Vitamin D: The relationship between alcohol consumption and vitamin D levels has yielded inconsistent findings. Although our study suggests that vitamin D supplementation may improve mental health outcomes, more standardized research is needed to understand the broader health implications of alcohol’s effects on vitamin D status [52].

Amlodipine: Amlodipine, primarily used to treat hypertension and angina, may alleviate anxiety symptoms in patients with PTSD indirectly. While not specifically indicated for mental health disorders like PTSD or AUD, its ability to address the associated physiological symptoms could enhance existing treatments [53].

Levothyroxine: Levothyroxine, used to treat hypothyroidism, may also support mental health by stabilizing mood and improving cognitive function. Our study highlights the importance of addressing alcohol-induced gut dysfunction, pro-inflammatory cytokines, and thyroid dysregulation in this patient population, suggesting that Levothyroxine could be beneficial for these patients [54].

Artenimol: Artemisinin (AR), a traditional Chinese medicine, has shown promise in reducing PTSD-like symptoms and chronic alcohol-induced liver injury in animal models. It may improve anxiety, cognition, and social behavior while protecting against alcohol-related liver damage, possibly through NF-κB inhibition [55,56].

Sulfasalazine: Originally used to treat inflammatory bowel diseases, Sulfasalazine may help to reduce the neuroinflammation and oxidative stress associated with PTSD and chronic alcohol consumption. It has been shown to reduce alcohol cravings and promote abstinence by inhibiting TNF-α and modulating oxidative stress markers. Sulfasalazine’s anti-inflammatory properties could alleviate alcohol-induced neuroinflammation and oxidative damage, supporting alcohol addiction treatment [57,58].

Glucagon-like peptide-1 (GLP-1) receptor agonists (Exenatide and Semaglutide): Clinical trials have shown that Exenatide and Semaglutide may reduce alcohol consumption and cravings in AUD patients, with Exenatide showing promise in altering brain activity associated with drug reward and addiction [59]. Using a retrospective chart review, patients with positive Alcohol Use Disorder Identification Test (AUDIT) scores (greater than 8) were identified and their scores were compared before and after Semaglutide treatment. The results showed a mean AUDIT score decrease of 9.5 points (*p* < 0.001) post-treatment. However, the results have been mixed, and gastrointestinal adverse events are common. Despite these challenges, GLP-1 receptor agonists remain a promising area of research for AUD treatment [60].

Other drugs: Additional medications mentioned in Table 2 may also reduce the risk of adverse events in PTSD + AUD patients, although more research is needed to assess their real-world effectiveness in this context.

### 4.2. Leveraging Advanced Predictive Models to Reduce Adverse Outcomes in PTSD and AUD Patients

Our study aimed to mitigate adverse events in these patients by employing data-driven models. We utilized both structured and unstructured EMR data, alongside non-EMR data, to develop a novel assessment tool to evaluate the risk of adverse events in this vulnerable population. By integrating information on drugs and their targets and employing systems pharmacology and deep learning techniques, we identified potential medications and proposed mechanisms for their effects. Through the use of EMR data, we also evaluated protein targets and their associated medications, enhancing the model’s predictive accuracy. Key strengths of our approach include the large sample size, the incorporation of multi-dimensional data, and the practical applicability of our algorithm in clinical practice. This comprehensive approach not only deepens the understanding of the biological mechanisms involved but also lays the groundwork for the identification of reliable biomarkers and potential pharmacotherapies for this high-risk group.

### 4.3. Limitations

Our study has several limitations. Firstly, due to the relatively small number of medications prescribed to patients, we were unable to assess the effects of drugs that were infrequently used or not used at all. This may have resulted in missing potentially important drug–outcome associations. In our study, we defined cases as patients who experienced an adverse event within three months of the index date, while controls had no adverse events during the same period. Although this conservative approach helped to clearly differentiate the groups, we recognize that some individuals may experience adverse events without developing PTSD. Additionally, our study did not account for the directionality of modulations or establish causative relationships, so any associations identified should not be interpreted as causal. Another limitation is the potential variability in PTSD diagnoses, as both the ICD-9 and ICD-10 criteria were used over the course of the study. The shift from the ICD-9 to ICD-10 in 2015 may have introduced inconsistencies in case identification, potentially affecting our results. Although we performed subgroup analyses and sensitivity tests that showed no significant impact from these diagnostic changes, differences in the criteria may still have influenced the findings. For future research, we plan to use multiple datasets and refine our algorithms. We will also aim to identify key biomarkers and leverage advanced deep learning models to analyze complex datasets, which could improve the accuracy of predictive models and enable more targeted interventions in the management of PTSD and AUD.

## 5. Conclusions

Our approach has the potential to significantly improve prevention efforts and reduce the impact of adverse events in these patients. In this study, we identified a range of medications that could help to mitigate the adverse event risks in this population, including OnabotulinumtoxinA (Botulinum Toxin A), Dronabinol, Acamprosate, Celecoxib, Exenatide, Semaglutide, Melatonin, Verapamil, vitamin D, Amlodipine, Levothyroxine, Artenimol, and Sulfasalazine. The insights provided by T-DeepBiomarker enable data-driven decision-making, allowing for the development of personalized prevention and intervention strategies tailored to high-risk PTSD + AUD patients.

## Figures and Tables

**Table 1:** The performance of T-DeepBiomarker.

**RETAIN(+SDOH)**	**1**	**2**	**3**	**4**	**5**	**Average**	**Standard Deviation**
Validation Data: AUC	0.962	0.964	0.956	0.956	0.965	0.961	0.004
Test Data: AUC	0.954	0.952	0.953	0.951	0.96	0.954	0.004
Test Data: Precision	0.912	0.886	0.904	0.903	0.931	0.907	0.016
Test Data: Recall	0.889	0.907	0.896	0.899	0.881	0.894	0.010
Test Data: F1	0.9	0.897	0.9	0.901	0.905	0.901	0.003
**LR(+SDoH)**	**1**	**2**	**3**	**4**	**5**	**Average**	**Standard Deviation**
Validation Data: AUC	0.878	0.874	0.875	0.866	0.862	0.871	0.007
Test Data: AUC	0.868	0.856	0.86	0.851	0.846	0.856	0.008
Test Data: Precision	0.75	0.7	0.696	0.671	0.696	0.703	0.029
Test Data: Recall	0.823	0.864	0.899	0.917	0.863	0.873	0.036
Test Data: F1	0.785	0.774	0.784	0.775	0.771	0.778	0.006
**TLSTM(+SDoH)**	**1**	**2**	**3**	**4**	**5**	**Average**	**Standard Deviation**
Validation Data: AUC	0.96	0.963	0.966	0.964	0.962	0.963	0.002
Test Data: AUC	0.941	0.953	0.96	0.961	0.958	0.955	0.008
Test Data: Precision	0.849	0.896	0.866	0.887	0.884	0.876	0.019
Test Data: Recall	0.903	0.884	0.929	0.914	0.916	0.909	0.017
Test Data: F1	0.875	0.89	0.896	0.9	0.899	0.892	0.010

Abbreviations: AUC: area under curve, TLSTM: Time-Aware Long Short-Term Memory, RETAIN: Reverse Time Attention model, LR: logistic regression, and SDoH: social determinants of health.

**Table 2:** Significant drugs and associated targets identified for prediction of adverse events.

Feature Name	Protein Name	Relative Contribution	Wilcoxon *p* Value	FDR-Q Non-Parametric Test	Bonferroni *p* Value	DrugBank ID	Drug Name
ASMT_HUMAN	Acetylserotonin O-methyltransferase	0.682	2.15 × 10^−12^	1.94 × 10^−10^	6.60 × 10^−9^	DB01065	Melatonin
PERE_HUMAN	Peripherin	0.656	2.66 × 10^−11^	1.74 × 10^−9^	8.17 × 10^−8^	DB01065	Melatonin
RORB_HUMAN	Nuclear receptor ROR-beta	0.680	8.03 × 10^−10^	3.85 × 10^−8^	2.47 × 10^−6^	DB01065	Melatonin
CNR1_HUMAN	Cannabinoid receptor 1	0.198	1.08 × 10^−9^	3.34 × 10^−8^	3.11 × 10^−6^	DB00470; DB00486; DB02955; DB05077; DB05201; DB05750; DB06155; DB09061; DB09288; DB11745; DB11755; DB14009; DB14011	Dronabinol, Nabilone, Ricinoleic acid, SLV319, V24343, Drinabant, Rimonabant, Canabidiol, Propacetamol, Otenabant, Tetrahydrocanabivarin, Medical Cannabis, Nabiximols
CNR2_HUMAN	Cannabinoid receptor 2	0.198	1.08 × 10^−9^	3.34 × 10^−8^	3.11 × 10^−6^	DB00470; DB00486; DB02955; DB06202; DB09061; DB11755; DB14009; DB14011; DB16321	Dronabinol, Nabilone, Ricinoleic acid, Lasofoxine, Tetrahydro-canabivarin, Medical Cannabis, SLV319, Canabidiol, Nabiximols
CA2D3_HUMAN	Voltage-dependent calcium channel subunit alpha-2	0.756	4.63 × 10^−9^	1.65 × 10^−7^	1.42 × 10^−5^	DB00153; DB00228; DB00421; DB00622; DB00661; DB09235; DB11148; DB13746	Ergocalciferol, Enflurane, Spironolactone, Nicardipine, Verapamil, Efonidipine, Butamben, Bioallethrin
CA2D3_HUMAN	Voltage-dependent calcium channel subunit delta-3	0.756	4.63 × 10^−9^	1.65 × 10^−7^	1.42 × 10^−5^	DB00153; DB00228; DB00421; DB00622; DB00661; DB09235; DB11148; DB13746	Ergocalciferol, Enflurane, Spironolactone, Nicardipine, Verapamil, Efonidipine, Butamben, Bioallethrin
VTDB_HUMAN	Vitamin D-binding protein	0.721	2.44 × 10^−7^	5.77 × 10^−6^	0.00075	DB00169	Vitamin D
ASM_HUMAN	Sphingomyelin phosphodiesterase	0.765	1.32 × 10^−6^	2.53 × 10^−5^	0.00405	DB00381; DB00477; DB01151; DB14009	Amlodipine, Chlorpromazine, Desipramine, Cannabis
SNP25_HUMAN	Synaptosomal-associated protein 25	0.203	2.12 × 10^−6^	2.42 × 10^−5^	0.00610	DB00083; DB16820	BotulinumtoxinA, LetibotulinumtoxinA
RHOB_HUMAN	Rho-related GTP-binding protein RhoB	0.203	2.12 × 10^−6^	2.42 × 10^−5^	0.00610	DB00083	BotulinumtoxinA
ITAV_HUMAN	Integrin alpha-V	0.783	3.94 × 10^−6^	6.40 × 10^−5^	0.0121	DB00451; DB16515	PLN-74809, Levothyroxine
H3BUU9_HUMAN	Cadherin 11	0.476	4.13 × 10^−6^	6.63 × 10^−5^	0.0127	DB00482	Celecoxib
GRM5_HUMAN	Metabotropic glutamate receptor 5	0.233	5.37 × 10^−6^	5.66 × 10^−5^	0.0154	DB00659; DB05070; DB06201; DB12733	Acamprosate, ADX10059, Rufinamide, Dipraglurant
GLP2R_HUMAN	Glucagon-like peptide 2 receptor	0.374	2.86 × 10^−5^	0.000315	0.0877	DB00040; DB08900	Teduglutide, Glucagon
TBB5_HUMAN	Tubulin beta-5 chain	0.182	0.000121	0.000760	0.347	DB00361; DB00541; DB00570; DB01179; DB01394; DB01873; DB03010; DB05147; DB05284; DB06042; DB06137; DB09130; DB11638; DB11641; DB11731; DB12334; DB15534	Vinorelbine, Vincristine, Vinblastine, Podofilox, Colchicine, Epothilone Ds, Patupilone, CYT997, CA4P, ZEN-012, Tirbanibulin, Copper, Artenimol, Vinflunine, Depatuxizumab, Milataxel, Colchicine
MAP4_HUMAN	Microtubule-associated protein 4	0.418	0.000183	0.00107	0.525	DB01229; DB01248; DB11638	Paclitaxel, Docetaxel, Artenimol
OX1R_HUMAN	Orexin receptor type 1	0.398	0.00799	0.0237	1	DB09034; DB11951; DB15031	Daridorexant, Lemborexant, Suvorexant
XCT_HUMAN	Myosin-2	0.284	0.00146	0.00565	1	DB00138; DB00142; DB00740; DB00795; DB06151; DB11590	Cystine, Glutamic Acid, Riluzole, Sulfasalazine, Acetyl Cysteine, Thimerosal
GLP1R_HUMAN	Cystine/glutamate transporter	0.625	0.00616	0.0226	1	DB00040; DB01276; DB06655; DB09043; DB09045; DB09265; DB13928; DB14027; DB15171; DB15650; DB16697	Glucagon, Exenatide, Liraglutide, Albiglutide, Dulaglutide, Lixisenatide, Semaglutide, Taspoglutide, Tirzepatide, Efpeglenatide, Glutazumab

## Data Availability

The original contributions presented in the study are included in the article; further inquiries can be directed to UPMC.

## Appendix A. Diagnosis Codes (See References [15,16])

### Appendix A.1. PTSD

309.81, F43.10, F43.11, F43.12.

### Appendix A.2. ASUD

291.0, 291.1, 291.2, 291.3, 291.4, 291.5, 291.8, 291.81, 291.82, 291.89, 291.9, 292.0, 292.11, 292.12, 292.2, 292.81, 292.82, 292.83, 292.84, 292.85, 292.89, 292.9, 357.5, 425.5, 535.30, 535.31, 571.0, 571.1, 571.2, 571.3, 648.30, 648.31, 648.32, 648.33, 648.34, 965.00, 965.01, 965.02, 965.09, 968.5, 969.6, E850.0, E854.1, E860.0, E935.0, E938.5,E939.6,V654.2, 303, 303.0, 303.00, 303.01, 303.02, 303.03, 303.9, 303.90, 303.91, 303.92, 303.93, 304, 304.0, 304.00, 304.01, 304.02, 304.03, 304.1, 304.10, 304.11, 304.12, 304.13, 304.2, 304.20, 304.21, 304.22, 304.23, 304.3, 304.30, 304.31, 304.32, 304.33, 304.4, 304.40, 304.41, 304.43, 304.5, 304.50, 304.51, 304.52, 304.6, 304.60, 304.61, 304.62, 304.63, 304.7, 304.70, 304.71, 304.72, 304.73, 304.8, 304.80, 304.81, 304.82, 304.83, 304.9, 304.90, 304.91, 304.92, 304.93, 305, 305.0, 305.00, 305.01, 305.02, 305.03, 305.1, 305.10, 305.12, 305.13, 305.2, 305.20, 305.21, 305.22, 305.23, 305.3, 305.30, 305.31, 305.33, 305.4, 305.40, 305.41, 305.42, 305.43, 305.5, 305.50, 305.51, 305.52, 305.53, 305.6, 305.60, 305.61, 305.62, 305.63, 305.7, 305.70, 305.71, 305.72, 305.73, 305.8, 305.80, 305.81, 305.83, 305.9, 305.90, 305.91, 305.92, 305.93, F10.10, F10.11, F10.120, F10.121, F10.129, F10.14, F10.151, F10.159, F10.180, F10.188, F10.19, F10.20, F10.21, F10.220, F10.221, F10.229, F10.230, F10.231, F10.232, F10.239, F10.24, F10.250, F10.251, F10.259, F10.26, F10.27, F10.280, F10.288, F10.29, F10.920, F10.921, F10.929, F10.94, F10.951, F10.959, F10.96, F10.97, F10.980, F10.982, F10.988, F10.99, F11.10, F11.11, F11.120, F11.121, F11.129, F11.14, F11.159, F11.188, F11.19, F11.20, F11.21, F11.220, F11.221, F11.222, F11.229, F11.23, F11.24, F11.250, F11.259, F11.288, F11.29, F11.90, F11.921, F11.929, F11.93, F11.94, F11.988, F11.99, F12.10, F12.11, F12.121, F12.122, F12.129, F12.150, F12.151, F12.159, F12.180, F12.188, F12.19, F12.20, F12.21, F12.220, F12.23, F12.250, F12.259, F12.288, F12.29, F12.90, F12.920, F12.921, F12.922, F12.929, F12.959, F12.980, F12.988, F12.99, F13.10, F13.11, F13.129, F13.14, F13.180, F13.188, F13.19, F13.20, F13.21, F13.220, F13.221, F13.229, F13.230, F13.231, F13.232, F13.239, F13.24, F13.259, F13.27, F13.280, F13.29, F13.90, F13.920, F13.921, F13.929, F13.930, F13.931, F13.939, F13.94, F13.97, F13.980, F13.99, F14.10, F14.11, F14.120, F14.121, F14.122, F14.129, F14.14, F14.151, F14.159, F14.180, F14.182, F14.188, F14.19, F14.20, F14.21, F14.220, F14.221, F14.222, F14.229, F14.23, F14.24, F14.250, F14.251, F14.259, F14.280, F14.282, F14.288, F14.29, F14.90, F14.920, F14.921, F14.929, F14.94, F14.951, F14.959, F14.980, F14.988, F14.99, F15.10, F15.11, F15.121, F15.129, F15.14, F15.159, F15.180, F15.188, F15.20, F15.21, F15.220, F15.222, F15.229, F15.23, F15.259, F15.29, F15.90, F15.920, F15.921, F15.929, F15.93, F15.94, F15.950, F15.951, F15.959, F15.980, F15.982, F15.988, F15.99, F16.10, F16.11, F16.129, F16.159, F16.20, F16.21, F16.221, F16.24, F16.259, F16.283, F16.90, F16.921, F16.929, F16.950, F16.959, F16.980, F16.983, F16.988, F16.99, F17.200, F17.201, F17.203, F17.208, F17.209, F17.210, F17.211, F17.213, F17.218, F17.219, F17.220, F17.223, F17.228, F17.229, F17.290, F17.298, F17.299, F18.10, F18.11, F18.19, F18.20, F18.24, F18.90, F19.10, F19.11, F19.120, F19.121, F19.129, F19.14, F19.150, F19.159, F19.180, F19.181, F19.188, F19.19, F19.20, F19.21, F19.221, F19.229, F19.230, F19.231, F19.232, F19.239, F19.24, F19.259, F19.280, F19.29, F19.90, F19.920, F19.921, F19.922, F19.929, F19.930, F19.931, F19.939, F19.94, F19.950, F19.951, F19.959, F19.96, F19.980, F19.982, F19.988, F19.99.

### Appendix A.3. SRE

V62.84, R45.851, E950.3, E956, E950.4, E950.0, E958.8, T14.91, E950.9, E958.9, T14.91XA, E950.5, E950.2, E953.0, E958.1, E953.8, E950.1, E950.7, E952.0, E958.0, E957.1, E957.0, E958.5, E952.1, E955.4, T14.91XD, E950.6, E953.9, E955.0, E957.9, E958.7, E958.3, E954, T14.91XS, E951.0, E951.8, E952.8, E953.1, E955.1, E958.6, E958.2, E955.9, E955.2, X83.8XXA, T42.42A, T43.592A, T39.1X2A, X78.8XXA, X78.9XXD, T42.6X2A, X78.9XXA, T43.222A, T50.902A, X83.8XXD, T39.312A, T43.212A, T45.0X2A, X78.1XXA, X78.8XXD, T50.992A, T40.2X2A, T43.292A, T43.012A, T39.012A, T42.8X2A, X78.0XXA, T51.0X2A, T40.5X2A, T40.4X2A, T40.1X2A, T44.7X2A, T38.3X2A, T44.6X2A, T48.1X2A, T46.5X2A, T71.162A, T48.3X2A, T43.022A, T44.3X2A, T50.902D, X79.XXXA, T65.92XA, X78.1XXD, T51.92XA, T42.1X2A, T65.892A, T56.892A, T43.622A, X80.XXXA, T42.4X2D, X78.0XXD, T42.72XA, T43.3X2A, X76.XXXD, T48.4X2A, T51.2X2A, T46.4X2A, T39.1X2D, T40.7X2A, T54.92XA, T40.602A, T45.512A, X76.XXXA, T43.222D, T39.392A, T47.1X2A, T50.902S, X74.9XXD, T39.092A, T38.1X2A, X74.9XXA, T39.312D, T38.892A, T43.612A, X82.8XXA, T42.6X2D, T43.632A, T46.1X2A, T45.0X2D, T50.992D, T54.2X2A, T40.3X2A, T39.012D, T43.4X2A, T58.02XA, T43.592D, X81.0XXA, T43.202A, T43.8X2A, T44.992A, T45.2X2A, T40.1X2D, T41.292A, T50.2X2A, T48.6X2A, T50.7X2A, T49.0X2A, T46.3X2A, T42.0X2A, T36.1X2A, T36.0X2A, X74.9XXS, X72.XXXD, T43.012D, T51.8X2A, T51.0X2D, T54.92XS, T54.3X2A, T65.892D, T65.92XD, T65.222D, T50.3X2A, T48.5X2A, T47.0X2A, T46.6X2A, T65.92XS, T54.1X2A, T52.4X2A, T52.0X2A, T55.1X2A, T59.892A, T42.4X2S, T42.3X2A, T36.3X2A, T37.8X2A, T38.2X2A, T38.3X2D, T40.992A, T40.8X2A, T44.4X2A, T43.692A, T45.2X2D, T44.7X2D, T43.502A, T71.192A, X79.XXXD, X83.2XXA, X83.8XXS, X72.XXXA, X71.9XXA, T48.202A, T40.2X2D, X74.8XXS, T48.3X2D, T39.8X2A, T47.4X2A, T47.6X2A, T50.6X2A, T49.6X2D, T43.3X2D, T50.5X2A, X74.01XA, X73.0XXA, T49.6X2A, X72.XXXS, X78.9XXS, T39.92XA, X80.XXXD, X81.8XXA, T39.4X2A, X77.8XXA, T50.2X2D, T43.622D, T43.292D, T45.4X2A, T46.0X2A, T41.3X2A, T42.5X2A, T42.6X2, T46.7X2A, T46.8X2A, T46.5X2D, T43.1X2A, T43.92XA, T40.5X2D, X71.0XXS, X71.3XXA, X71.8XXA, T43.212D, T46.2X2A, T40.8X2D, T40.602D, T43.022D, T44.1X2A, T46.4X2D, T65.222S, T62.0X2A, T71.162D, T51.1X2A, T51.2X2D, T51.2X2S, T52.8X2A, T51.92XD, T50.8X2A, T56.892D, T58.92XA, T54.3X2S, T54.3X2D, T54.0X2A, T55.0X2A, T36.0X2D, T36.4X2A, T38.5X2A, T36.8X2A, T37.5X2A.
